# Supplementary material for: Complexome profiling of the Chlamydomonas psb28 mutant reveals TEF5 as an early PSII assembly factor
Source: Plant Cell. 2025 Mar 17;37(6):koaf055. doi: 10.1093/plcell/koaf055 (PMC12164590; doi:10.1093/plcell/koaf055)
Supplement: koaf055_Supplementary_Data [file koaf055_supplementary_data.zip › psb28_interactive.zip!/plots/intensity/qualityPlots/peptides/_Cre01.g042200.t1.1.html]

### Cre01.g042200.t1.1

Trivial name:   
SeqCoverage [median]: 13.10  
Mass: 10.14 kDa  
  
 

| Sequence | MissedCleavage | Mass [Da] | wtIntensity | muIntensity | Score |
| --- | --- | --- | --- | --- | --- |
| APLDKPTTPGK | 1 | 1123.62 | 37976.91 | 53341.15 | 160.36 |
| QKAPLDKPTTPGK | 2 | 1379.78 | 7748.85 | 4453.65 | 110.92 |
|  |  |  | 45725.8 | 57794.8 |  |
